# 

**Published:** 1894-08

**Authors:** 


### TABLE OF CONTENTS.

ORIGINAL COMMUNICATIONS. PAGE

Some Observations on the Extraction of Teeth to prevent Decay. By EUGENE H. SMITH, D.M.D., Boston, Mass. . . . . . . . . . . . . 481
An Interesting Case of Exostosis; Asepsis in Operations. By ALBERT WESTLAKE, D.D.S., New York . . . . . . . . . . . . . . . . . 485
Massage in Rheumatism of the Temporo-Maxillary Articulation and Muscles of the Lower Jaw. By DOUGLAS GRAHAM, M.D., Boston, Mass. . . 487
On Some Relations of Diseases of the Nose and Throat to Dentistry. By JOHN W. FARLOW, M.D., Boston, Mass. . . . . . . . . . . . . . . 491
Homœopathic Treatment of Toothache from Pulp-Capping. By CHARLES H. TAFT, D.M.D., Chicago, Ill. . . . . . . . . . . . . . . . . . . . 495
The Dental Pulp, Abscesses, and Root-Filling. By CHARLES KEYES, D.D.S , Rio de Janeiro, Brazil . . . . . . . . . . . . . . . . . . 499
Further Remarks on Pyorrhœa Alveolaris. By C. N. PEIRCE, D.D.S., Philadelphia . . . . . . . . . . . . . . . . . . . . . . . . 501
A Reply to Dr. Kirk's Article on Coagulation. By MAX GREENBAUM, D.D.S., Philadelphia . . . . . . . . . . . . . . . . . . . . . . 509

REPORTS OF SOCIETY PROCEEDINGS.

American Academy of Dental Science . . . . . . . . . . . . . . . . . 514
Harvard Odontological Society . . . . . . . . . . . . . . . . . . . 524
Society of Alumni, University of Pennsylvania . . . . . . . . . . . 527
Connecticut State Dental Association . . . . . . . . . . . . . . . . 528

EDITORIAL.

Principle in Practice . . . . . . . . . . . . . . . . . . . . . . . 530

OBITUARY.

Dr. J. W. Rhone . . . . . . . . . . . . . . . . . . . . . . . . . . 532

DOMESTIC CORRESPONDENCE.

Reply to Editorial in the *Dental Cosmos* . . . . . . . . . . . . . 533

NOTES AND COMMENTS . . . . . . . . . . . . . . . . . . . . . . . . 536

CURRENT NEWS.

Woman's Dental Association of the United States . . . . . . . . . . 539
Recent Patents . . . . . . . . . . . . . . . . . . . . . . . . . . . 540

SELECTIONS.

Need of Independent Medical Journals . . . . . . . . . . . . . . . . 543
Is Lysol Poisonous? . . . . . . . . . . . . . . . . . . . . . . . . 543

